# Instrumental broadening and the radial pair distribution function with 2D detectors

**DOI:** 10.1107/S2053273324006569

**Published:** 2024-07-15

**Authors:** Dmitry Chernyshov, Kenneth P. Marshall, Erlend Tiberg North, Chloe A. Fuller, David S. Wragg

**Affiliations:** a Swiss–Norwegian Beamlines at the European Synchrotron Radiation Facility, 38000 Grenoble, France; b Centre for Materials Science and Nanotechnology, Department of Chemistry, University of Oslo, PO Box 1033, Blindern, 0315 Oslo, Norway; c Institute for Energy Technology IFE, Norway; Columbia University, USA

**Keywords:** powder diffraction, instrumental resolution, pair distribution functions, 2D detector

## Abstract

The atomic pair distribution function (PDF) is a real-space representation of the structure of a material. Experimental PDFs are obtained using a Fourier transform from total scattering data which may or may not have Bragg diffraction peaks. The determination of Bragg peak resolution in scattering data from the fundamental physical parameters of the diffractometer used is well established, but after the Fourier transform from reciprocal to direct space, these contributions are harder to identify. Starting from an existing definition of the resolution function of large-area detectors for X-ray diffraction, this approach is expanded into direct space. The effect of instrumental parameters on PDF peak resolution is developed mathematically, then studied with modelling and comparison with experimental PDFs of LaB_6_ from measurements made in different-sized capillaries.

## Abstract 2

The instrumental resolution function for pair distribution functions (PDFs) with 2D detectors is expressed and used to simulate the impact of various parameters on experimental PDF data sets.

## 1. Introduction

The instrument resolution function – a fundamental characteristic of any diffractometer – describes the peak profile measured and its angular dependence, due to the specific geometry and conditions of that particular diffractometer. It is routinely characterized with standard, highly crystalline samples and, in many cases, it can also be calculated theoretically (Caglioti *et al.*, 1958 ▸; Gozzo *et al.*, 2006 ▸) or modelled with ray-tracing simulations (Rebuffi *et al.*, 2017 ▸). Any diffraction pattern is then a convolution of the scattering function from the sample and the instrumental resolution function for the diffractometer on which it was measured. The accurate decoupling of these contributions is important for structural and microstructural refinement from Rietveld and pair distribution function (PDF) analyses of powder diffraction data.

With the benefits of low noise, high dynamic range, high efficiency of detection and fast read-out, experimental setups based on large-area photon-counting detectors with thick Si or CdTe sensors are extensively used for total scattering measurements at synchrotron sources and are becoming more common in the home laboratories. The separation of sample and instrument contributions to the resulting data is straightforward in reciprocal space and the known resolution function can be used (Neder & Proffen, 2020 ▸). Analysis of interatomic distances in direct space, on the other hand, requires a Fourier transform of the diffraction intensity, resulting in the PDF. The Fourier transform of the diffraction pattern gives a product of the Fourier transforms of the resolution and scattering functions. The simplest possible instrumental resolution function is a single Gaussian broadening, which is trivial to model as its Fourier transform results in an envelope function that exponentially decays with interatomic distance. However, the situation becomes more complicated if we have to account for the *Q* dependence of the parameters of the resolution function, in particular for the change of full width at half-maximum (FWHM) as a function of the scattering vector.

A simple Gaussian *Q*-dependent resolution function has been proposed for which an analytical solution to the real-space data has been found. The FWHM of the diffraction peaks, 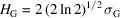, depends on *Q* as 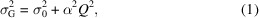
where 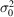 is the variance of the *Q*-independent broadening component summing up instrumental and sample-related contributions and α is a constant encompassing the instrumental parameters for a certain diffractometer (Qiu *et al.*, 2004 ▸). This solution was first presented by Thorpe *et al.* (2002 ▸) and was recently revisited and derived for Lorentzian and pseudo-Voigt profiles (Beyer *et al.*, 2022 ▸), but crucially the resolution function for a measurement with large-area detectors was recently reported (Chernyshov *et al.*, 2021 ▸), and has a very different *Q* dependence to that in equation (1).

Here we consider the effect of the resolution function for such experimental setups on the PDF data. First, we re-express the resolution function as a function of scattering vector and compare it with a standard diffraction pattern collected on a large-area detector. Further analysis is done within the theoretical scheme proposed by Thorpe *et al.* (2002 ▸). Second, we compare diffraction patterns measured with a powdered LaB_6_ standard using different sets of instrument parameters. Finally, we evaluate corresponding PDF data and illustrate the effect of the resolution function numerically.

## 2. Theory

The instrumental broadening of diffraction lines for a large 2D detector oriented normal to the beam is derived by Chernyshov *et al.* (2021 ▸). The square of the instrumental FWHM as a function of diffraction angle, 2Θ, is given by 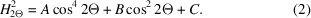
Hereinafter we assume the angular uncertainty is the variance of a normal distribution. The coefficients in equation (2) are defined by the experimental parameters as follows: 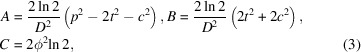
where *D* is the sample-to-detector distance, *p* is the pixel size (or point spread function, if any) of the detector, *t* is the thickness of the detector’s sensitive layer, *c* is the size of the sample (*e.g.* the diameter of the capillary with a powder sample) and ϕ denotes the angular divergence of the scattered beam.

### 2.1. The resolution function in *Q* space

Consider a Gaussian centred at *Q*′ in *Q* space: 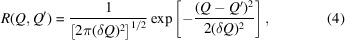
where (δ*Q*) is the variance. The angular uncertainty of the corresponding Bragg angle reads (Chernyshov *et al.*, 2021 ▸)
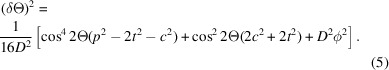
Given that 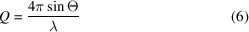
and 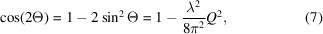
we can write 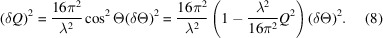
With 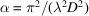 and 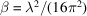 the variance of the angular resolution function becomes 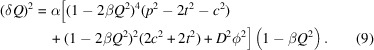
Alternatively, the variance as a function of *Q* can be written as the tenth-order even polynomial function: 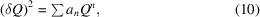
with the coefficients expressed via components of the resolution associated with sample size, beam divergence, sample-to-detector distance and detector parameters – pixel size and thickness of the sensitive layer (see Appendix *A*[App appa]).

**Figure 1:**
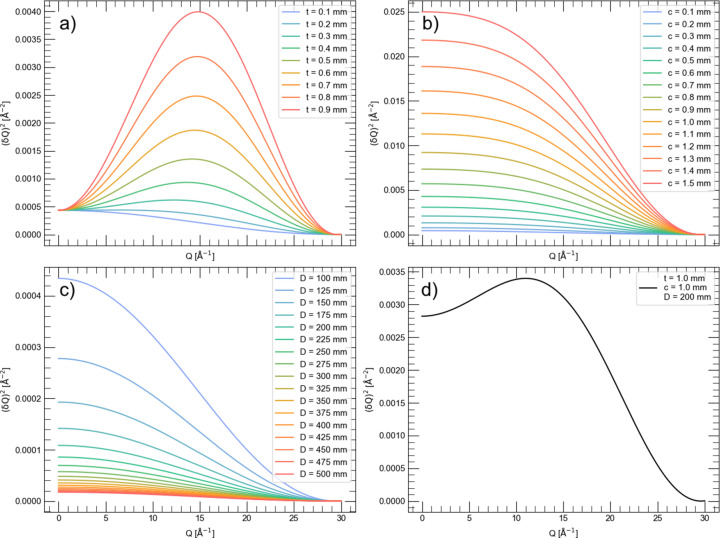
Calculated resolution functions for changing experimental parameters of a simulated diffraction experiment. (*a*) shows the effects on change in detector sensitive layer thickness (*t*) with a capillary size (*c*) of 0.1 mm and a sample-to-detector distance (*D*) of 100 mm. (*b*) varies *c* with *t* = 0.1 mm and *D* = 100 mm. (*c*) showcases *D* with *c* = 0.1 mm and *t* = 0.1 mm. Subplot (*d*) presents a calculated resolution function given a typical experimental setup with a 1.0 mm capillary situated 200 mm from a detector with a sensitive layer thickness of 1.0 mm.

The effect of some instrumental parameters on (δ*Q*)^2^ is shown in Fig. 1 ▸. It is clear that the default assumption from equation (1) does not hold for measurements with a large-area detector set normal to the incoming beam. At *Q* = 0, (δ*Q*)^2^ = *a*_0_ = α(*p*^2^ + *c*^2^ + *D*^2^ϕ^2^). There is also a high-angle/high-*Q* limit of (δ*Q*)^2^ = 0 when the scattered X-ray is normal to the incoming beam and parallel to the detector surface. Therefore, 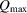 < 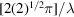 and 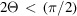.

### 2.2. The resolution function in PDF *r* space

Following the considerations suggested by Billinge & Thorpe (2002 ▸), we can derive the effect of instrumental broadening on the PDF. Assuming the Gaussian approximation holds, the observed radial PDF, *G*_*c*_(*r*), can be expressed using the following convolution: 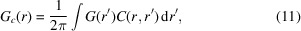
where *G*(*r*′) is the actual radial PDF and *C*(*r*, *r*′) is a transmission function, *i.e.* the real-space instrumental contribution.

*C*(*r*, *r*′) can be expressed as the Fourier transform of the resolution function in *Q* space [equation (4)]: 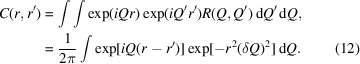
While there is no analytical solution, this integral can be evaluated numerically with the polynomial expression for variance [equation (10)] and the coefficients listed in Appendix *A*[App appa]. Equation (12) can be written in the following form:
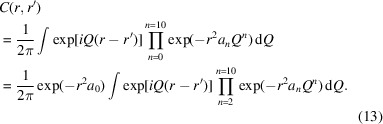
The exponent in front of the integral is a damping function that suppresses the PDF at high *r*. Note that the damping parameter is a function of various experimental parameters: 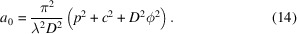
The integral expresses the position-dependent broadening of PDF features, while the damping parameter, 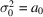, sets a limit on the size of the structural correlations or structural coherence, *i.e.* particle size, that can be probed for certain experimental conditions. Using an intensity threshold of 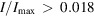 [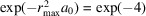] to denote the decay of signal to only noise defines the maximum interatomic distance that is measurable: 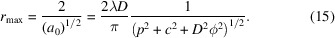
For a detector with size, *V*, in any direction perpendicular to the incoming beam (the aperture of the detector), the detectable scattering vectors are ≤*Q*_max_, where 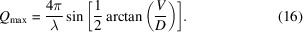
Therefore, for a detector with fixed position and aperture, we can define the relation 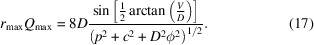
For a typical detector with vertical size 300 mm and pixel size 0.172 mm, positioned 150 mm from the sample, a wavelength of 0.3 Å would correspond to a *Q*_max_ of 22 Å^−1^.

Given a capillary diameter and beam size of 0.3 mm and neglecting beam divergence, one gets 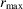 = 83 Å; therefore, for the given experimental conditions, it is not possible to reliably determine particle sizes larger than 8 nm. Increasing the capillary and beam size to 0.7 mm decreases *r*_max_ to 4 nm. Obviously, a non-zero beam divergence will further reduce *r*_max_, and an increase in accuracy (signal–noise ratio) will lead to higher *r*_max_.

The shape and width of PDF features are given by the integral in equation (13). It is easy to see that, in contrast to the resolution expected by default [equation (1)], the decrease in width of diffraction lines with *Q* does not result in a Gaussian as derived by Thorpe *et al.* (2002 ▸). The function 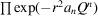 in equation (13) is now limited by *Q*_max_, and the corresponding Fourier transform may be approximated by a sinc(*rQ*_max_) function, at least for small *r*′. We investigate the broadening by numerical calculation of the resolution function as a function of *r* for a set of *r*′ values and find that it is rather insensitive to the actual shape of the resolution function. This conjecture is illustrated in Appendix *B*[App appb]; it implies that there is effectively no instrumental position-dependent broadening in the PDF pattern.

We have therefore split instrumental broadening of Bragg lines into two components: a term that leads to a decay of the intensity of the PDF pattern with interatomic distance [equation (14)], and a *Q*-dependent part that results in a broadening of PDF peaks. The first component sets a limitation on the maximal size of coherent regions [equation (14)]. The second component defines instrumental contribution in the width of PDF peaks, which is predominantly defined by 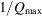. For the majority of synchrotron experiments, the detector and beam size are fixed while the sample-to-detector distance and X-ray wavelength may be varied; characteristic dependencies of *r*_max_ and *Q*_max_ are shown in Appendix *C*[App appc].

The above derivations assume a high degree of monochromatization of the incoming beam. However, it becomes common practice to increase the bandwidth δλ to have higher intensity, which adds the following term to equation (5) (Chernyshov *et al.*, 2021 ▸): 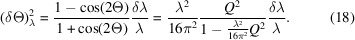
The corresponding contribution in the line broadening in *Q* space, as follows from equation (8), is written 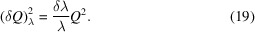
Therefore, the effect of increased bandwidth is an increase of the *a*_2_ coefficient [see equation (10) and Appendix *A*[App appa]], that affects the width of PDF features but does not reduce the maximal size of a structurally coherent region. Since the bandwidth contribution is always positive, in contrast to the other coefficients in the series equation (10), we can consider the final broadening as a convolution of a Gaussian [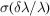] with a sinc function (Appendix *B*[App appb]). Therefore, the effect of a broad bandwidth will dominate for measurements done up to very high *Q*.

We conclude the theoretical part with a comment on the geometry where the detector is tilted by an angle α to cover a larger angular range. The corresponding expression for the resolution as a function of diffraction angle is derived by Chernyshov *et al.* (2021 ▸): 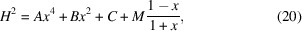
where *x* = 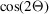 or *x* = 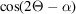 and *M* = 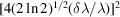, and the last term takes energy bandwidth into account [equation (18)]. For such a geometry the resolution function in *Q* space can be evaluated numerically.

## 3. Experimental illustration of resolution effects

Powdered LaB_6_ samples were measured at the BM31 beamline (Swiss–Norwegian Beamlines at the European Synchrotron Radiation Facility, Grenoble, France) using a MAR345 2D detector with a wavelength of 0.270793 Å, at a distance of 181 mm and a maximum *Q* of 23 Å^−1^. The sample was loaded in various sizes of glass capillary (0.1, 0.2, 0.3, 0.4, 0.5 and 0.7 mm in diameter). Corresponding backgrounds were measured using the same size capillaries and were subtracted from the monitor-normalized and integrated patterns. Calibration of the instrument and integration of images were done using *pyFAI* (Ashiotis *et al.*, 2015 ▸). The FWHMs of individual Bragg lines as a function of *Q* are given in Appendix *E*[App appe] and are rather scattered at high *Q* due to strong overlap of Bragg lines; however, the trend corresponds well to the expected instrumental resolution [equations (2), (10), see also Fig. 1 ▸].

**Figure 2:**
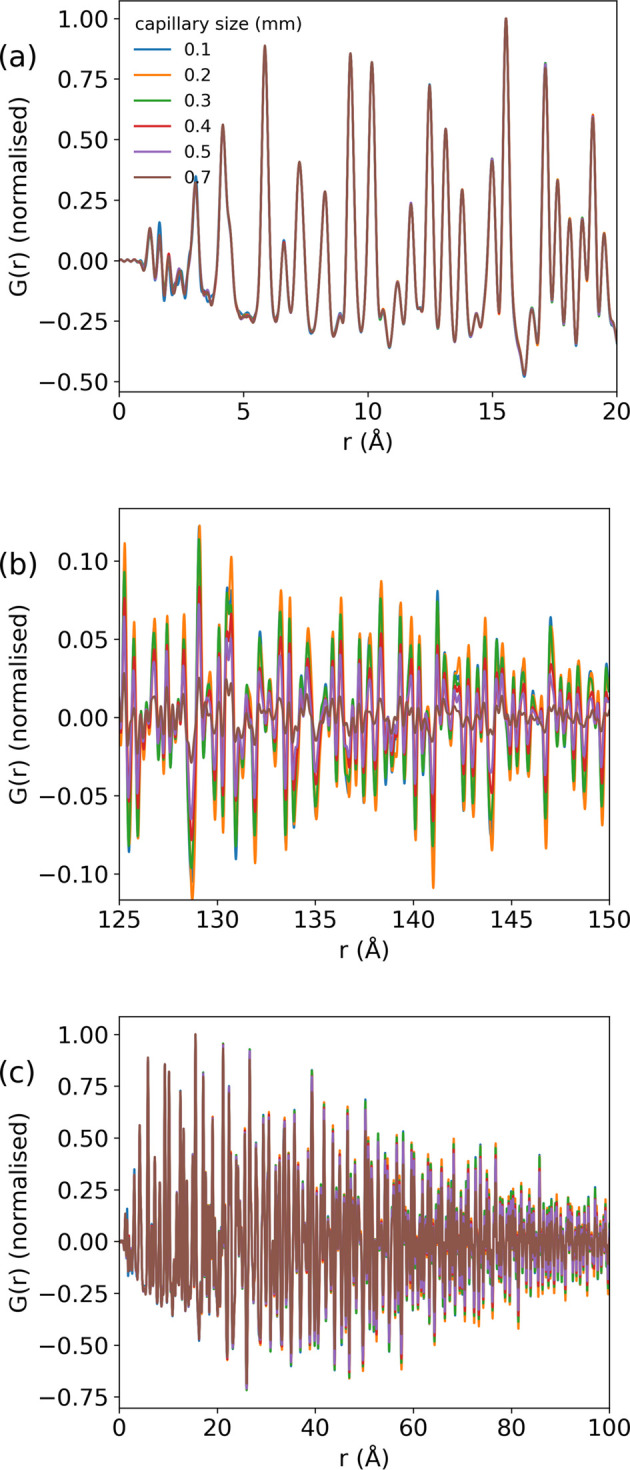
Experimental PDF patterns (normalized to 1) of LaB_6_ measured in 0.1, 0.2, 0.3, 0.4, 0.5 and 0.7 mm capillaries plotted to (*a*) 20 Å, (*b*) 125–150 Å and (*c*) 100 Å.

*PDFgetX3* was used to convert the 1D total scattering patterns to total scattering structure functions [*S*(*Q*) and reduced form, *F*(*Q*)] and PDF [*G*(*r*)] (Juhás *et al.*, 2013 ▸). The PDFs are shown in Fig. 2 ▸ with different *r* ranges (normalized to the maximum intensity). With 0.7 mm capillaries the signal decays to 2% of the maximum at 120 Å {converting the pattern to an approximate *g*(*r*) function, *G*(*r*) = 4πρ_0_*r*[*g*(*r*) − 1], the signal decays to 2% at 75 Å}. The lattice parameter, appropriate atomic positions, damping and atomic displacement parameters were refined against these PDFs using *Diffpy-CMI* (Juhás *et al.*, 2015 ▸). The damping was refined using a combined Gaussian–Lorentzian profile as described by Beyer *et al.* (2022 ▸). The Lorentzian component for the 0.1 mm capillary refined to 0.007, and this fixed value was used for all the other refinements, while the Gaussian component was freely refined. The results of the refinements are shown in Appendix *D*[App appd], Figs. 7–9, with the *R*_p_ values shown in Table 1 ▸.

**Figure 3:**
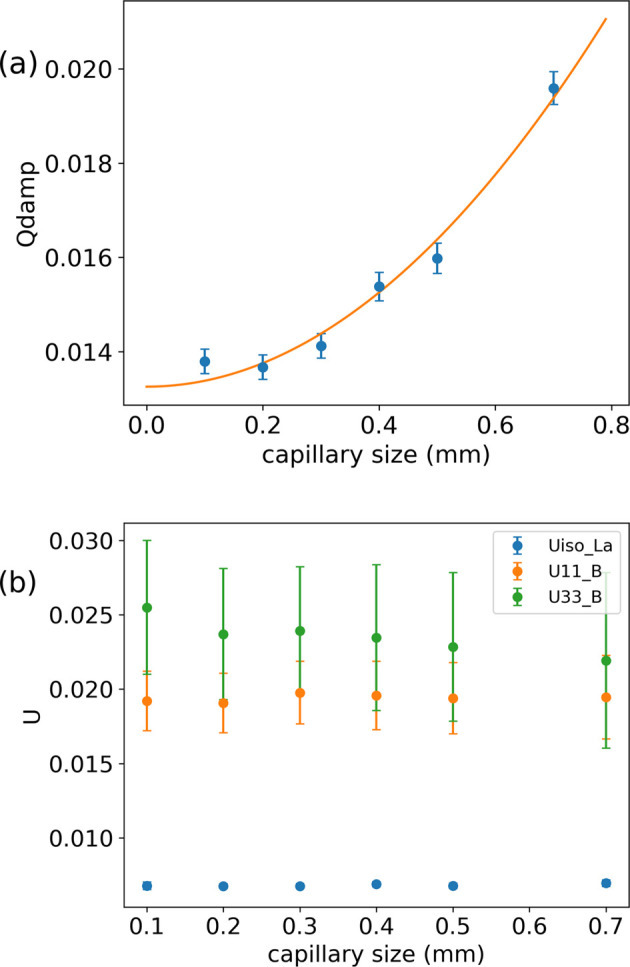
(*a*) *Q*_damp_ with a quadratic fit and (*b*) *U* atomic displacement parameters refined for the PDF patterns for each capillary size.

**Figure 7:**
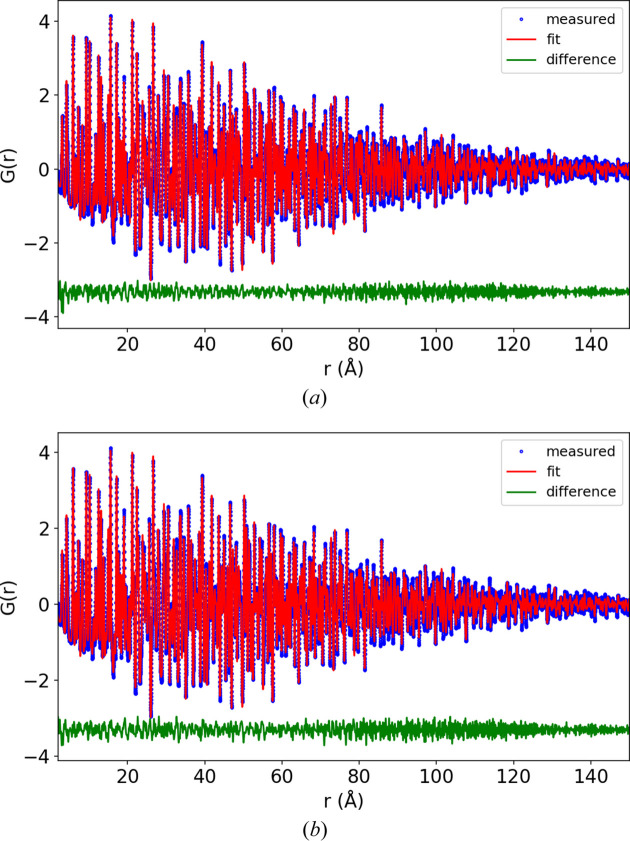
LaB_6_ refinements for (*a*) 0.1 and (*b*) 0.2 mm capillaries.

**Figure 8:**
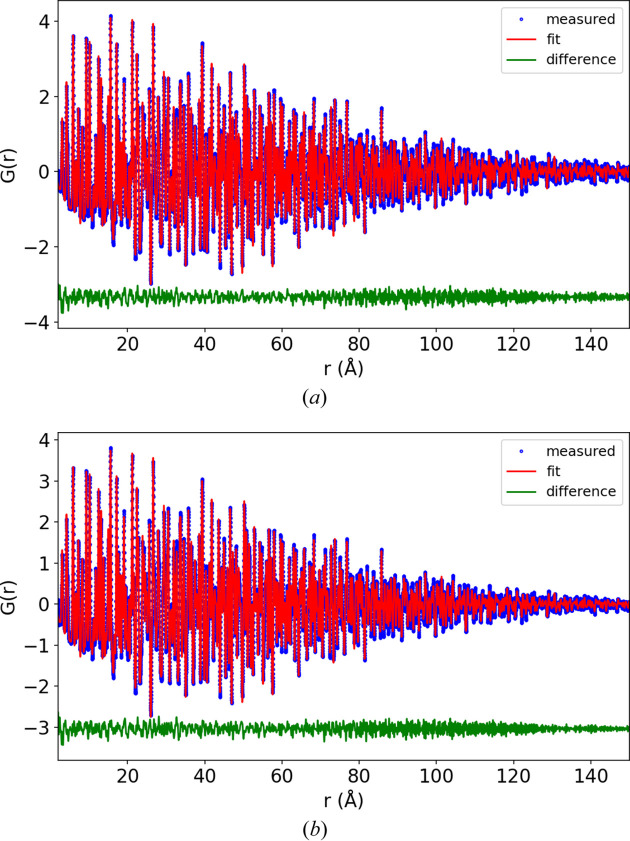
LaB_6_ refinements for (*a*) 0.3 and (*b*) 0.4 mm capillaries.

**Figure 9:**
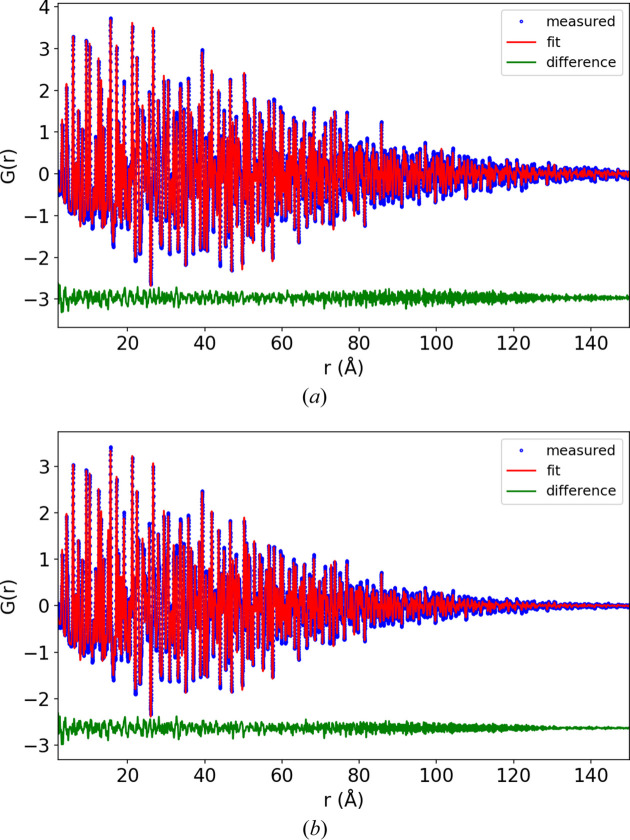
LaB_6_ refinements for (*a*) 0.5 and (*b*) 0.7 mm capillaries.

The refined *Q*_damp_ and *U* atomic displacement parameters are shown in Fig. 3 ▸. The largest impact of capillary size is, predictably, on *Q*_damp_, while peak broadening is much less affected, as shown by the smaller effect on the atomic displacement parameters. Although the refined value for *U*_33_ for boron appears to reduce with capillary size (0.025 to 0.022 going from the 0.1 to 0.7 mm capillaries), this trend is within the error of the refinement. *Q*_damp_ as a function of capillary size was fitted with a quadratic function, the result of which was *Q*_damp_ = 0.0133 + 0.0125*c*^2^, in agreement with equation (14). The small impact of capillary size on the broadening in total scattering patterns can also be seen visually in Fig. 2 ▸(*a*) by observing that in the range 0–20 Å in *r*, the PDF profiles appear to match almost exactly.

## 4. Conclusions

We considered the instrumental line broadening for the case when powder diffraction of monochromatic radiation detected by a large-area detector is used for analysis of the PDF. We have shown that for a detector set normal to the incident beam, the broadening of PDF peaks predominantly depends on the maximal *Q* value reached in the experiment and to a much lesser extent on the instrumental broadening of the diffraction lines.

We have also derived the instrumental damping of the PDF signal as a *Q*-independent contribution to instrumental broadening defined by sample/beam size, pixel size of the detector and beam divergence. The overall instrumental contribution to the width of Bragg lines leads to a decay of PDF intensity with interatomic radius: thus the broader the lines in the diffraction pattern, the smaller the particle sizes that can be analysed by the PDF method. The corresponding analytical expression is derived and given by equation (14), with a graphical illustration shown in Appendix *C*[App appc]. The practical importance of the derived results may be better illustrated by the following set of experimental parameters: λ = 0.2 Å, sample-to-detector distance is 300 mm, Pilatus 2M detector with pixel size 0.172 mm and beam/capillary size 0.3 mm. The setup offers *Q*_max_ = 33 Å, which seems to be good for a generic PDF experiment; however the PDF intensity will be damped down at 5 nm by the instrumental resolution irrespective of the actual particle sizes. The diffraction experiment may therefore be planned, from the resolution point of view, as an optimizing trade-off between the damping of PDF intensity and the width of PDF peaks.

The analytical expressions derived here are based on a series of approximations, such as a symmetric Gaussian line shape, for the sake of simplicity. Experimental data collected with a set of geometrical conditions confirm the expected effects of the instrumental broadening. Nevertheless, accurate numerical simulations with more realistic line shapes are necessary to improve the estimates presented here.

## Figures and Tables

**Figure 4:**
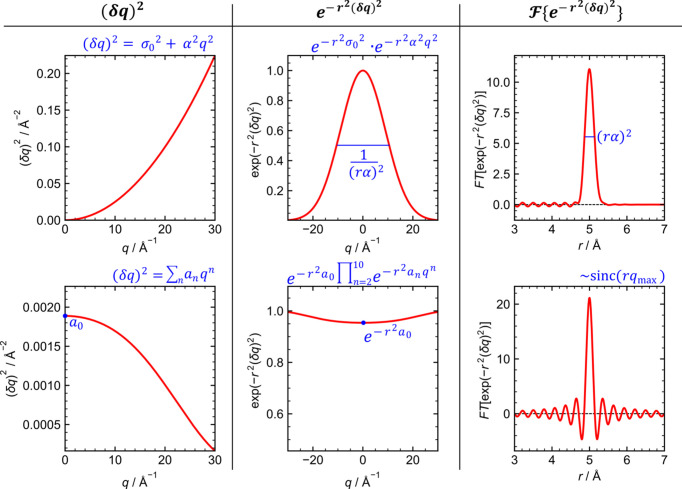
Comparison between the different resolution functions described in equations (1) and (10). Left-hand column: the instrumental resolution function (FWHM broadening as a function of *q*). Middle column: exponential function in equation (12). Right-hand column: Fourier transform of the exponential function. Calculations were performed using the following parameters: *Q*_max_ = 20 Å^−1^, *D* = 100 mm, λ = 0.25 Å, *p* = 0.172 mm, *c* = 0.3 mm and *t* = 0.1 mm.

**Figure 5:**
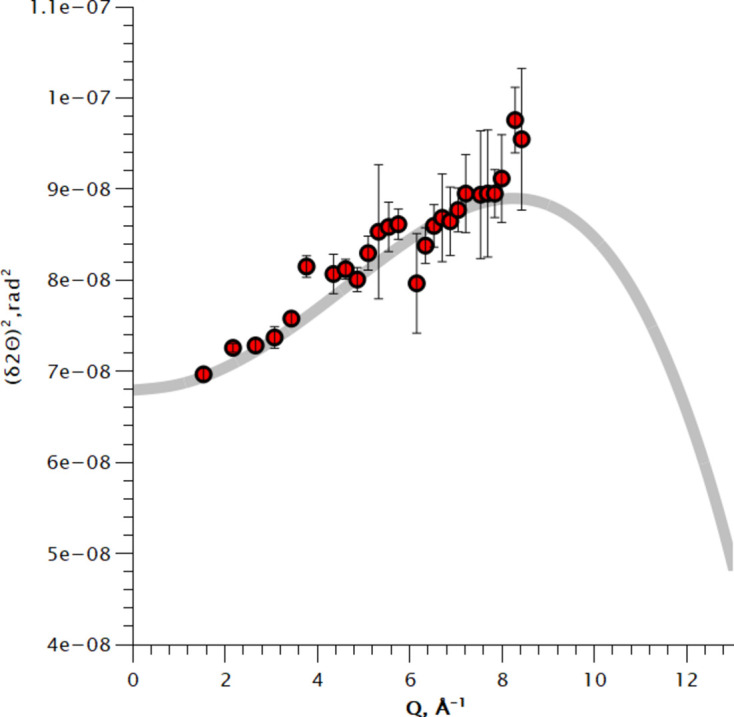
Experimental instrumental resolution at *D* = 890 mm, Pilatus2M 1 mm CdTe detector. Circle markers show the FWHM of diffraction lines of LaB_6_, and the grey line shows the model calculation.

**Figure 6:**
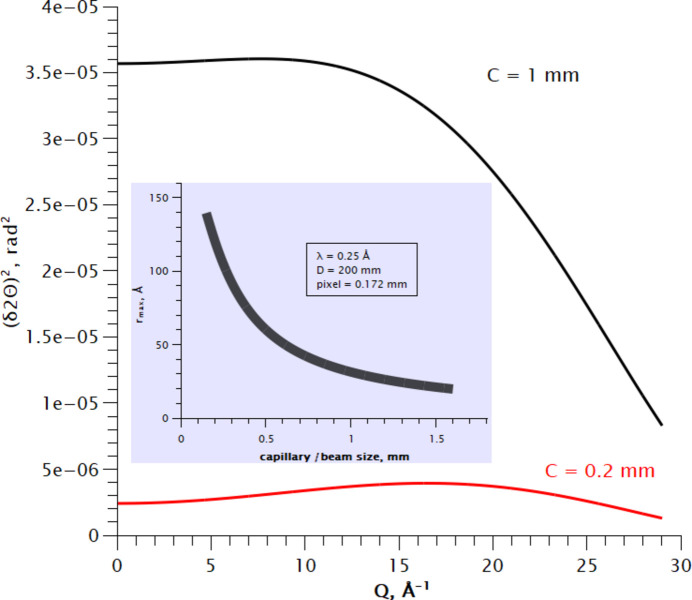
Calculated instrumental resolution at *D* = 200 mm, Pilatus2M 1 mm CdTe detector for small and large capillary sizes. Inset: *r*_max_ as a function of capillary size.

**Figure 10:**
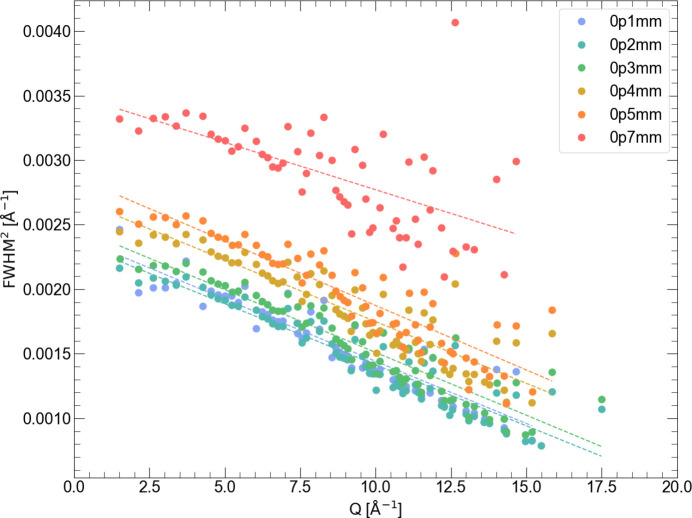
FWHM of LaB_6_ peaks for capillary sizes 0.1 to 0.7 mm from Gaussian peak fitting, filtering out peaks with low intensity. Each data set was filtered to a linear regression to clarify the trends at high *Q*.

**Figure 11:**
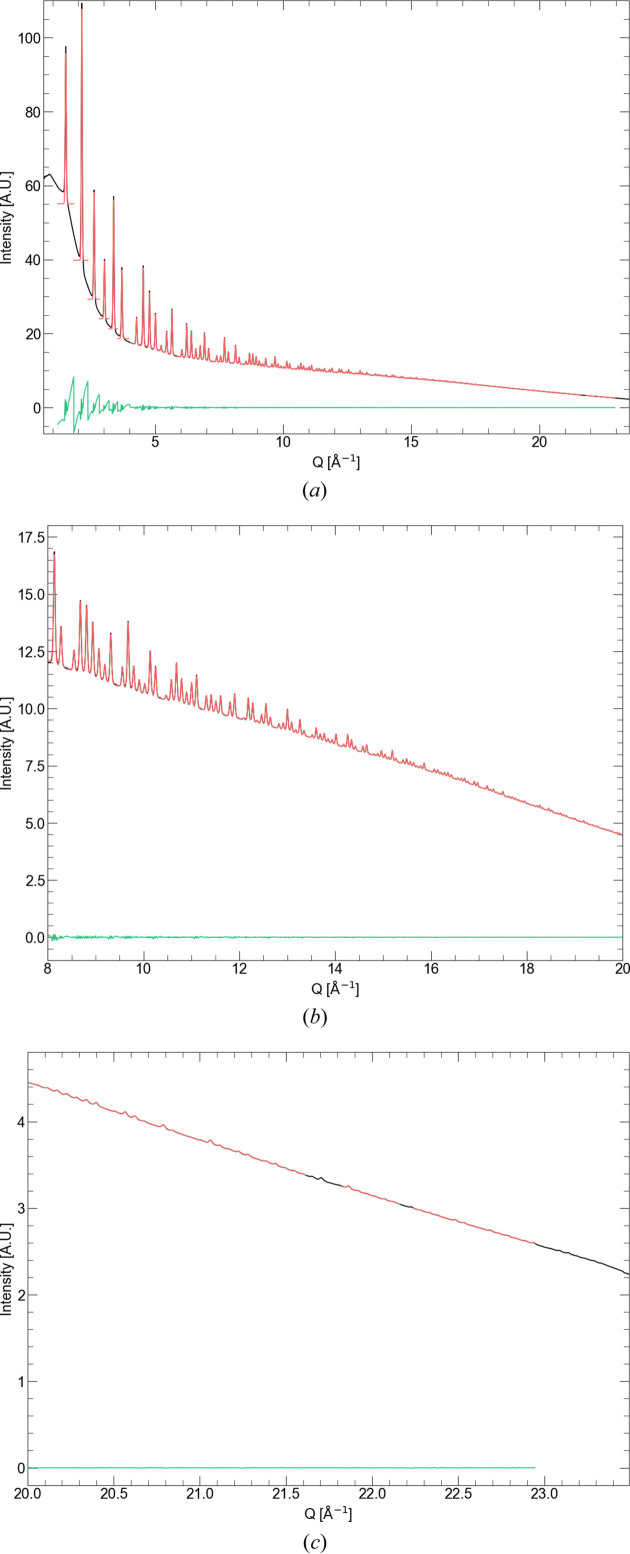
Observed (black), calculated (red) and difference (green) plots for the fitting of the X-ray diffraction data from the 0.1 mm capillary.

**Table 1:** *R*_p_ values for the PDF refinements for each capillary size

	Capillary size (mm)					
	0.1	0.2	0.3	0.4	0.5	0.7
*R*_p_	0.11	0.14	0.11	0.12	0.11	0.11

## Appendix A

Let us denote  and . The coefficients in equation (10) are

## Appendix B

Fig. 4 ▸ shows a comparison between the different resolution functions described in equations (1) and (10).

## Appendix C

The experimental resolution function in *Q* and 2Θ space is shown in Fig. 5 ▸.

The test measurement was done at the BM31 beamline with a Pilatus 2M detector with 1 mm of CdTe sensitive layer at 890 mm distance from the sample. The wavelength (0.25 Å), sample-to-detector distance (890 mm), pixel size (0.172 mm) and capillary size (0.2 mm) were fixed in the model; the effective thickness of the sensitive layer, size of the beam and divergence correction were refined. The beam was focused on the detector which gives negative divergence correction and a larger spot at the sample position. The effective thickness depends on the wavelength (Chernyshov *et al.*, 2021 ▸); for the present case it is 0.38 mm. This value was used to illustrate the resolution function for a parallel beam reduced to the diameter of the sample capillary in conditions for PDF measurements. Two limiting cases and an estimate of *r*_max_ are shown in Fig. 6 ▸.

## Appendix D

The results of the refinements are shown in Figs. 7 ▸, 8 ▸, 9 ▸, Table 1 ▸.

## Appendix E Validation of theory through experimental measurements of LaB_6_ with different capillary sizes

Experimental X-ray diffraction data collected on BM31 for 0.1–0.7 mm internal diameter capillaries were fitted using Python and the *Scipy* module’s curve fitting. Each peak was fitted with an independent Gaussian function (refined position, intensity, FWHM, background) to obtain the FWHM peak height. The peaks were fitted from the midpoint of the previous peak to the midpoint of the next peak. A fifth-order Chebyshev polynomial fitted to the background in *TOPAS V6* was subtracted from the data prior to peak fitting. The FWHMs plotted against *Q* in Fig. 8 ▸ clearly resemble the calculated functions in Fig. 1 ▸ (capillary size). The data shown are filtered to show the points where the relative peak intensity is 5% or more above the background, as the low signal levels at high *Q* values led to a large number of outliers which obscured the overall trends. The final fits for the 0.1 mm capillary are shown in Fig. 9 ▸. The fitting method assumes the background between peaks to be linear, and despite the initial background correction this leads to some artefacts in the difference plot at low *Q*. The effect of these on the FWHM values from the Gaussian fits is negligible (see Figs. 10 ▸, 11 ▸).
